# Supplementary figures and images for: SARS-CoV-2-Specific Immune Responses in Vaccination and Infection during the Pandemic in 2020–2022
Source: Viruses. 2024 Mar 13;16(3):446. doi: 10.3390/v16030446 (PMC10974545; doi:10.3390/v16030446)

# Epidemic waves of SARS-CoV-2 infection in Japan

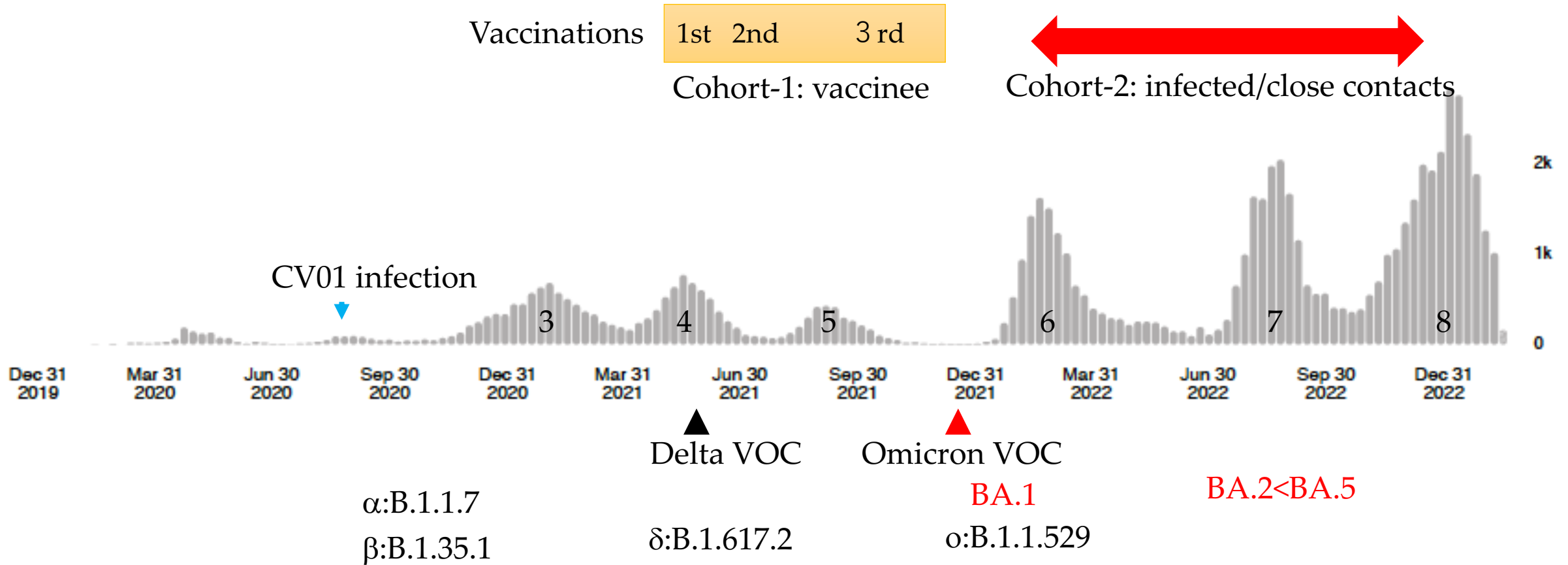

Supplement: Supplementary file 1 [file viruses-16-00446-s001.zip › viruses-2878000-supplementary.pdf]
